# Role of Anti-MICA Antibodies in Graft Survival of Renal Transplant Recipients of India

**DOI:** 10.1155/2018/3434050

**Published:** 2018-04-05

**Authors:** Mohit Chowdhry, R. N. Makroo, Mandhata Singh, Manoj Kumar, Yogita Thakur, Vandana Sharma

**Affiliations:** Transplant Immunology, Sarita Vihar, Delhi Mathura Road, Molecular Biology and Transfusion Medicine Apollo Hospitals, New Delhi 110076, India

## Abstract

**Introduction:**

The MIC (MHC class I chain-related) genes are a group of nonclassical MHC genes, located in the MHC class 1 region of chromosome 6. The aim of the present study was to find the prevalence of MHC class 1 chain-related (MICA) alloantibodies in patients undergoing live-related donor renal transplantation and its role in short-term graft survival. The role of blood transfusion in the formation of these antibodies was also studied.

**Materials and Methods:**

Pretransplant samples of patients undergoing renal allograft transplantation were tested for anti-MICA antibodies. Association of various demographics, HLA-A + B + DRB1 mismatches, anti-HLA antibody screen, and anti-MICA antibodies was assessed using Pearson's chi-square test.

**Results:**

Out of 646 serum samples, 94 (14.6%) were positive and 552 (85.4%) were negative for anti-MICA antibodies. Patients with anti-MICA antibody had a graft survival 89.3% as compared to 94.7% in patients without anti-MICA antibody (*P* < 0.05). The hazard ratio for all patients was 3.0701 (*P* < 0.05). Out of the 340 patients with no HLA antibodies, the presence of anti-MICA antibodies without any HLA antibodies (*n* = 43) was associated with poor outcome in the patients (hazard ratio of 2.768, *P* < 0.05). The presence of MICA antibodies with HLA antibodies did not decrease the graft survival (hazards ratio of 1.3750, *P* > 0.05).

**Conclusion:**

Preformed MICA antibodies independently increase the risk of kidney rejection and therefore recommend that guidelines should be formed for mandatory testing of these antibodies prior to renal transplant.

## 1. Introduction

The MIC (MHC class I chain-related) genes are a group of nonclassical MHC genes, located within the MHC class 1 region of chromosome 6 [1]. MICA gene encodes synthesis of a stress-induced protein and is highly polymorphic. Nearly 15–36% of the 11 kb DNA of MICA has sequence homology with the classical HLA class 1 genes [2]. The MICA antigens act as ligands for the activating C-type lectin-like receptor (NKG2D) which is expressed on NK cells, *γδ* T cells, and CD8+ *αβ* T cells [3]. Interaction of MICA with NKG2D leads to activation of antigen-specific cytotoxic T-lymphocyte-mediated cytotoxicity, NK cell responses, and cytokine production. Besides, polymorphic MICA antigens are capable of inducing antibodies that may kill target cells in the presence of complement. Hence, MICA is involved in both the innate and adaptive immune responses [3]. Northern blotting of the MICA genes has revealed their expression on almost every organ in the human body with the exception of the central nervous system [4]. The presence of MICA antigens on the endothelium and their polymorphic behavior are associated with the presence of anti-MICA antibodies in the transplanted patients. This was confirmed by the study conducted by Zwirner and his colleagues, who tested the sera of transplanted patients against recombinant MICA proteins [3].

MICA is the most polymorphic nonclassical class I gene known so far with 105 alleles reported and new alleles being continuously identified [1]. Zou and colleagues [5] suggested that the immunogenicity of the MICA antigens is significant and the antibodies formed against these antigens can lead to antibody-mediated rejection (AMR) through complement-mediated cascade activation. However, Lemy and colleagues found better survival in patients positive for MICA antibodies [6]. Similarly, Solgi et al. [7] did not find significant difference in rejection episodes on comparing patients with or without the presence of anti-MICA antibodies.

The exact mechanism for the development of anti-MICA antibodies is unknown although alloimmunization through pregnancy and previous transplants has been reported. However, the role of blood transfusions in formation of anti-MICA antibody is not fully clear. There have been contrasting findings with regard to blood transfusions as a sensitizing event. Lemy et al. concluded that the blood transfusions, previous transplantation, and two or more pregnancy were significantly associated with the formation of anti-MICA antibodies [6]. However, the study of Zou et al. revealed that the blood transfusions were not implicated in the formation of anti-MICA antibodies [5].

Most studies on MICA antibody testing have been performed on deceased donors, and very limited literature is available for live-related donors especially in this part of the world. Also, to the best of our knowledge, this is the most extensive analysis of anti-MICA antibody testing by single-antigen bead (SAB) assay from Indian subcontinent.

The present study was undertaken with the following aims: firstly, to know the prevalence of pretransplant anti-MICA alloantibodies in patients undergoing live-related donor renal transplantation and its role in short-term graft survival and secondly, to determine if there is a role of blood transfusions in formation of anti-MICA antibodies.

## 2. Materials and Methods

The study was conducted in a tertiary care hospital of North India from June 2015 to December 2016. Six hundred forty-six, consecutive, first transplant recipients referred for pretransplant anti-MICA antibody assessment as a part of institutional protocol for transplant workup were included in our study.

Two different cohorts were included in the study. In cohort A (*n* = 646), the patients were divided into 2 groups based on the anti-MICA antibody test results, namely, MICA positive and MICA negative. Anti-HLA antibody screen, the patient-donor HLA antigen mismatch, hazards ratio, gender, and geographical history were compared between the two groups. The graft survival in both the groups was evaluated. The impact of 3-log leucoreduced-packed red cell transfusion on the formation of anti-HLA and anti-MICA antibody was also studied in this cohort.

In cohort B (*n* = 392), patients, in whom both anti-HLA antibody and anti-MICA antibody were performed, were divided as both anti-HLA antibody screening and anti-MICA antibodies positive (HLA+MICA+), both anti-HLA antibody screening and anti-MICA antibodies negative (HLA−MICA−), anti-MICA antibodies positive and anti-HLA antibody screen negative (HLA−MICA+), and anti-MICA antibodies negative and anti-HLA antibody screen positive (HLA + MICA−).

As HLA typing was possible in 304 patients, association of HLA A-B-DR mismatches with the pretransplant anti-MICA antibody positive and negative patients was studied in these patients.

DNA was extracted from peripheral leukocytes by DNA isolation kit (Invitrogen, USA PureLinkTM Genomic DNA Mini KitLot number 1822107) according to manufacturer's instruction, and DNA was diluted with 150 *μ*l elution buffer and stored at −20°C until further analysis. HLA typing was carried out by polymerase chain reaction sequence-specific primer (PCR-SSP) method (All Set+tm Gold SSP HLA-ABDR, Life Technologies, USA) utilizing allele-specific primers along with the control primers to identify the respective allele.

A freshly obtained, undiluted neat sera were used to perform all antibody assays. Analysis of anti-MICA antibody was done on Luminex platform by SAB assay (Lifecodes LSATM MIC, lot number 02077A-RUO, Immucor, USA). The beads are designed to detect IgG antibodies to MICA proteins (specificity for MICA^∗^001, ^∗^002, ^∗^004,^∗^005,^∗^006, ^∗^007, ^∗^008, ^∗^009, ^∗^011, ^∗^012, ^∗^015, ^∗^016, ^∗^017, ^∗^018, ^∗^019, ^∗^024, ^∗^028, ^∗^029, ^∗^030, ^∗^033, ^∗^036,^∗^037, ^∗^041, ^∗^042, ^∗^043, ^∗^046, ^∗^050, and ^∗^051). The conjugate comprised of antihuman globulin IgG secondary antibody (donkey F(ab')2) conjugated to phycoerythrin in a phosphate-based storage buffer containing NaCl, Tween-20, and sodium azide. For anti-MICA antibody, testing individual bead raw MFI value (>1000 MFI), background corrected MFI (BCM), background corrected ratio (BCR), and antigen density-BCR (AD-BCR) were calculated. As per manufacturer's protocol, the bead was considered positive if two or more of these adjusted values were above the cut-off values.

HLA antibody screening/panel-reactive antibody (PRA) test was based on the method of Luminex xMAP® Technology using the Lifecodes. Generally, based on controls, a raw MFI value <1000 was considered negative. In case the value of the control beads was high, suggesting background error (as neat sera were used), average adjusted MFI values were taken as the final value and interpreted accordingly.

The immunosuppression regimen remained the same for both anti-HLA antibody and anti-MICA antibody-positive patients, namely, triple immunosuppression of tacrolimus, mycophenolate mofetil, and corticosteroid with desensitization through rituximab and plasmaphereses with IVIG. All antibody-negative cases were managed with triple immunosuppression alone. Rejection was defined based on clinical parameters that include rising serum creatinine levels in the absence of other pathologies. All the rejection cases were biopsy proven and scored according to Banff ‘07 updated diagnostic criteria [8] with or without supplementary C4d staining on immunohistochemistry. Graft was considered as lost, upon return of patient to dialysis, or graft nephrectomy. The graft function was assessed and analyzed posttransplant on a regular basis, namely, 3 months, 6 months, and 12 months. Hazard ratio was calculated in patients of transplant rejection who had antibodies against MICA as compared to those who did not have these antibodies.

### 2.1. Statistical Analysis

All data was tabulated, and relevant parameters were statistically analyzed using the chi-square test. *P* < 0.05 was considered statistically significant. Kaplan-Meier survival plot and log-rank test (used to compare graft survival between patients with and without MICA antibodies) were performed using GraphPad Prism version 6.00 for Windows, GraphPad Software, La Jolla, California, USA, http://www.graphpad.com [9].

In addition, one-way multivariate analysis of variance (MANOVA) to test the hypotheses that there would be one or more mean difference between the factors (HLA antibody, MICA antibody and HLA mismatch) and graft survival was performed to check for the effect of potential confounders. Significance was assumed at *P* value less than 0.05. However, prior to conducting the MANOVA, a series of Pearson correlations were performed between all of the dependent variables in order to test the MANOVA assumption that the dependent variables would be correlated with each other in the moderate range. Statistical analysis was also performed using IBM SPSS Statistics software version 20.0 and R-3.2.1 (SPSS Inc., Chicago, IL, USA). The results were compared with the existing literature.

## 3. Results

In cohort A, out of 646 serum samples tested (466 (72%) males and 180 (27.8%) females) for anti-MICA antibodies, 94 (14.6%) were positive and 552 (85.4%) were negative. The demographic details of the patients in anti-MICA antibody-positive and anti-MICA antibody-negative patients are listed in Table 1. The effect of pretransplant, 3 log leucoreduced-packed red cell transfusions on formation of antibodies against HLA class I and class II or MICA, was also studied (Table 2).

Among 38 patients with antibodies against HLA class I, 33 patients (86.84%) received transfusions (*P* < 0.05) whereas out of the 608 recipients without any HLA class I antibodies, history of transfusion was present in 355 (58.4%) patients. Among 43 patients with antibodies against HLA class II, 38 patients (88.4%) received transfusions (*P* < 0.05) whereas out of the 603 recipients without any HLA class II antibodies, history of transfusion was present in 353 (58.5%) patients. In contrast to HLA antibodies, of the 94 patients with antibodies against MICA antigens, 9 (9.6%) patients received transfusions. Similarly, out of 552 anti-MICA antibody-negative patients, 68 (12.3%) patients received one or more blood or blood components and 484 (87.7%) patients did not receive transfusions (*P* > 0.05). Most of the blood donors were males (90%), and only 10% of the total donors were females.

In cohort B (Table 3), out of the total patients, testing for both anti-HLA and anti-MICA antibodies was possible in 392 patients. Anti-HLA antibody screening and anti-MICA antibodies were both negative in 297 (87.4%) patients and both positive in 8 (15.4%) patients. Anti-MICA antibodies positive, anti-HLA antibody screen negative was seen in 43 (12.6%) patients. Anti-MICA antibodies negative, anti-HLA antibody screen positive was seen in 44 (84.6%) patients. Patients with the presence of anti-MICA antibodies without any anti-HLA antibodies (*n* = 43) were associated with poor outcome in the patients (hazard ratio of 2.768, *P* < 0.05). The presence of anti-MICA antibodies with anti-HLA antibodies did not reduce the graft survival (hazard ratio of 1.3750, *P* > 0.05) (Table 4).

Influence of anti-MICA antibodies on graft survival in various subgroups like all patients, patients with HLA-A+B+DR mismatches, and anti-HLA antibody screen was studied (Table 4). Patients with anti-MICA antibody had a graft survival 89.3% as compared to 94.7% in patients without anti-MICA antibody (*P* < 0.05). The hazard ratio for all patients was 3.0701 (*P* < 0.05).

HLA typing and association of HLA-A-B-DR mismatches with the pretransplant anti-MICA antibody positive and negative patients was possible in 304 patients (Table 5). In 0-1 mismatches (*n* = 47), anti-MICA antibody was positive in 6 cases whereas it was negative in 41 cases. In 2–4 mismatches (*n* = 204), anti-MICA antibody was negative in 177 cases whereas it was positive in 27 cases. In 5-6 mismatches (*n* = 53), anti-MICA antibody was positive in 6 cases whereas it was negative in 47 cases. No clinically significant association of HLA mismatch with the presence of anti-MICA antibody and graft survival was seen.

**Figure 1:**
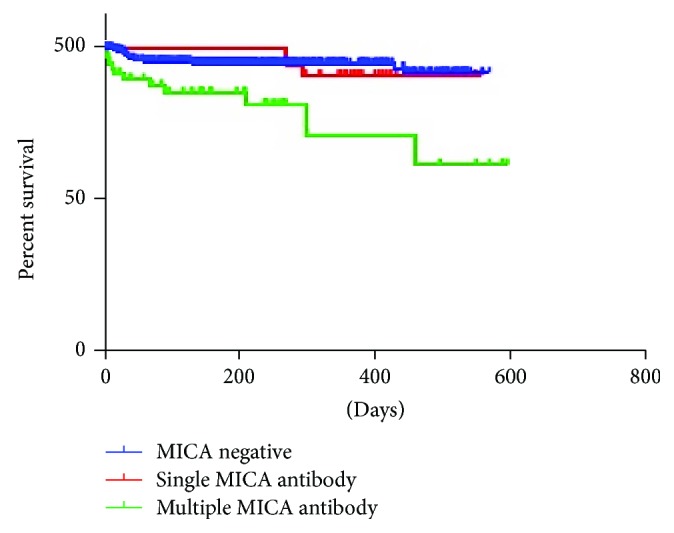
Graft survival in anti-MICA antibody.

The patients were also studied based on the number of anti-MICA antibodies produced, whether single or multiple antibodies (Table 6). Out of the 94 patients with anti-MICA antibody, 35 patients (24 males, 11 females) had antibody specific to a single MICA allele whereas 59 patients (29 males, 30 females) had antibodies positive to multiple MICA alleles. The average mean fluorescence intensity (MFI) was 2584 (range 1516 to 4805). In the multiple antibody groups, the average mean fluorescence intensity (MFI) was 4396 (range 1932 to 13,786). The most common antibody was anti-MICA^∗^041 (*n* = 34, 35.8%) antibody which was present either alone or with other anti-MICA antibodies. Comparison of graft survival between patients with no anti-MICA antibody and patients versus single antibody *versus* antibodies against multiple MICA antigens was compared and was found to be clinically significant (*P* < 0.05) (Figure 1). Multivariate analysis of variance (MANOVA) to test the hypotheses that there would be one or more mean difference between the factors (anti-HLA antibody, anti-MICA antibody, and HLA mismatch) and graft survival was performed. A statistically significant MANOVA effect was obtained, Pillai's trace = 0.448, F(3,642) = 1.740, *P* < 0.05.

## 4. Discussion

Although most of the graft failures are attributed to HLA antibodies, polymorphisms distinct from those of the HLA system may also affect the outcome of kidney allografts. Antibodies against endothelial cells were observed among many recipients in whom kidney allografts were rejected. Other polymorphisms possibly associated with kidney allograft failure are those involving vimentin, platelet-specific antigens, chemokines and their receptors, and molecules of the renin-angiotensin system. Evidence that HLA alone is not involved in graft rejection was provided by comparison of kidney transplant survival in patients with sibling donors and unrelated living donors wherein 38% of graft failure occurred due to other non-HLA antibodies [10]. Furthermore, Opelz in their study stated that chracterization of non-HLA antigens is much needed, as substantial number of graft failures in identical sibling donor transplants occurs due to these non-HLA antibodies [11]. According to Zou et al. [5], graft rejection in the renal recipients with HLA-matched donors and recipients who were not previously sensitized against HLA antigens is possible and is mostly attributed to non-HLA antibodies.

MICA antigens are expressed on the surface of the endothelial cells and are mostly found on the surface of the epithelial cells and fibroblasts [1] and keratinocytes and monocytes, but not on the surface of CD4+, CD8+, and CD19+ lymphocytes [12]. Therefore, the complement-dependent cytotoxicity (CDC) crossmatch, which employs the use of lymphocytes to detect the preformed antibodies, misses the antibodies present against the MICA antigens. Anti-MICA antibodies have been implicated in allografts undergoing both acute and chronic rejection. MICA binds to the C-type lectin receptor, NKG2D, leading to the secretion of cytokines that enhance the response of CD4+ T cells causing an augmented reaction thereby generating signals from the T cell to the B cell and triggering antibody production [13]. While the MICA antibodies present in the recipient plasma before the transplant can cause immediate graft rejection, the MICA antibodies formed after the antigen trigger provided by the transplant can initiate a process of damage and repair in the endothelium, thus leading to chronic rejection [14].

The presence of MICA antibodies in the plasma of a prospective renal transplant recipient can potentiate a graft rejection process unless measures to reduce the titers of the antibody are implemented. While certain researchers opine the MICA antibodies cannot bind complement [15], others suggest anti-MICA antibody can cause a C4d-positive AMR [16]. Álvarez-Márquez et al. [14] described donor-specific MICA antibodies in two of 19 renal allograft recipients with C4d-positive AMR. Cai et al. in their study on posttransplant sera concluded that only C1q-fixing antibodies were associated with graft failure which was related to AMR [17].

In the present study, out of 646 serum samples tested, 94 (14.6%) samples were found to be positive for MICA antibodies for one or more MICA alleles. A cohort of this data was presented as an abstract in the 43rd Annual Meeting of American Society for Histocompatibility and Immunogenetics [18]. Ozawa et al. in their study found that anti-MICA antibodies were found in 12% of total 266 patients and were found to be more frequent (21%) in patients with graft failure than in patients with successful graft (7%) [19]. Zou et al. [5], in an international collaborative study involving 20 centers in 13 countries on pretransplant samples found 217 out of 1910 patients (11.4%) had anti-MICA antibodies. They found a 1-year graft survival rate of 88.3 ± 2.2% as compared to 93.0 ± 0.6% among patients in the anti-MICA antibody-negative group (*P* = 0.01). Similarly, Zhang et al. [20] in their study discussed that the antibodies against MICA were positive in 15 out of the 52 patients (28.9%). A retrospective study involving 727 renal allograft recipients published by Sánchez-Zapardiel et al. [21] revealed a 7.15% prevalence of anti-MICA antibodies in patients waiting for a renal transplant.

The association of anti-MICA antibodies with a reduced allograft survival was evident from our study in which we observed 3.744 hazard ratio for all patients (*P* < 0.05). Many prior retrospective studies have also proven that donor-specific antibodies against MICA antigen are detrimental to the graft. For example, Sumitran-Holgersson et al. in a study on 139 renal allograft recipients in 2002 showed a significant correlation of anti-MICA antibodies with graft loss [22]. Zou et al. in their study found that the anti-MICA antibodies were found in elutes of kidneys, which had been rejected [4]. Three years later, Mizutani and coworkers in their retrospective study of “serial ten-year follow-up of HLA and MICA antibody production prior to kidney graft failure” found that anti-MICA antibodies were found in the sera of patients with graft failure at a higher frequency than in those with functioning grafts [23]. Furthermore, the association of antibodies against MICA and allograft rejection was highly significant in patients with no HLA antibodies indicating MICA antibodies as an independent risk factor in graft survival. This may explain for the allograft rejection in well-matched kidney donors with no HLA antibodies. Another study by the same authors indicated that 52% of recipients with graft rejection had anti-MICA antibodies compared with 21% of those with functioning grafts. (*P* < 0.001) [24]. These anti-MICA antibodies detected at pretransplant period could have a role in the development of AMR. Similarly, Zhang et al. [20] deliberated that the presence of MICA antibodies was associated with renal allograft deterioration as after one-year follow-up and the estimated glomerular filtration rate (eGFR) decreased (24.0 ± 3.4%) among recipients with anti-MICA antibodies compared to only (8.4 ± 3.0%) (*P* = 0.017) in patients without anti-MICA antibody. Narayan et al. [25] stated that donor-specific anti-MICA antibodies can be associated with both AMR and acute cellular reaction (ACR). They emphasized the need for early detection and screening for donor-specific MICA antibodies because these are not detected using our current crossmatch procedures. Furthermore, the serial quantification and monitoring of MICA MFI levels could alter the clinical management of allograft rejection and need to be assessed in large randomized, prospective trials. Sánchez-Zapardiel et al. [21] reported that preformed anti-MICA antibodies significantly increased the risk for allograft rejection particularly early after transplantation and that this effect was independent of the presence of anti-HLA antibodies. However, no significant difference was noticed in allograft survival and rejection rates at 2-year follow-up. Moreover, no significant epidemiological or clinical differences were observed between MICA antibody-positive and MICA antibody-negative groups. Their study did not define the donor specificity of anti-MICA antibodies. The same group of authors further demonstrated that presence of anti-MICA antibodies at pretransplant periods can bind native MICA molecules on the cell membrane and was able to mediate cell death by fixing and activating the complement cascade by using both the C1q single-antigen bead assay and complement-dependent cytotoxicity. In another study, the same group of authors concluded that the pretransplantation sensitization against MICA and HLA is an independent event. Furthermore, they stated that the preformed anti-MICA antibodies independently increase risk for kidney rejection and enhance the deleterious effect of PRA+ status early after transplantation [26].

In our study, the simultaneous presence of both anti-HLA antibody and anti-MICA antibody was quite rare (*n* = 8, 2.04%). Our findings corroborated well with the findings of Zou et al. [4], who found that the simultaneous presence of both these antibodies did not affect the graft outcome since this was a rare occurrence. In their 1910 patients tested for anti-HLA antibodies, only 1.9% of the patient had both MICA and HLA class I antibodies, and only 1.8% had anti-MICA and anti-HLA class II antibodies. This was in contrast to studies done by Ozawa et al. [19] who found that almost all patients with MICA antibodies also had HLA antibodies. Similarly, Panigrahi et al. [27] noted that the patients with graft rejection had either anti-HLA or anti-MICA antibody more frequently than patients with functioning graft. In fact, they could conclude that most of the times, both these antibodies coexisted together.

The HLA mismatch between the recipient and donor correlates well with the posttransplant rejection, but there has been a graft failure of some well-matched, low-risk kidney transplants patients' viz, recipients of first transplants, patients who received grafts from well HLA-matched donors, and recipients not previously sensitized against HLA. We tried to find out if the possible cause of rejection in these patients could be MICA antibodies. Zou et al. [5] in their study stated that the association of the MICA antibodies and the allograft rejection could be better observed in the patients who receive grafts well matched for HLA-A, HLA-B, and HLA-DR. They found allograft rejection with antibodies against MICA antigens is strong in better-matched cases without panel-reactive HLA antibodies. However, in our study, we did not find any significant association between the two variables.

In our study, the multivariate effect size was estimated at 0.448, which implies that 44% of the variance in the canonically derived dependent variable was accounted for by graft survival. A statistically significant MANOVA effect was obtained on analysis in the present study [28]. It meant that MICA antibodies are significantly and independently associated with reduced graft survival in donor graft, providing strong evidence for the involvement of these antibodies with graft rejection. Our findings corroborate well with the findings of the collaborative international study conducted by Paul Terasaki in deceased and living donors [29].

We had multiple patients with anti-MICA antibody specific to single MICA allele (anti-MICA^∗^041 antibody) with varying MFIs. However, in the absence of MICA-typing information, it is worthwhile to be cautious about the antibody that reacts with one allele. While reactivity with one allele is certainly possible, the certainty of it being true positive cannot be established. Since MICA^∗^041 is a relatively rare allele, the exact reason for the hypersensitization to the MICA^∗^041 allele in our cohort is unknown but is presumably because of prior sensitization from some infections and/or transfusions.

Our findings suggest that unlike anti-HLA antibodies, anti-MICA antibodies are not produced in response to the transfusion of blood or blood components and are uncommon in patients with a history of transfusion. Similarly, Zou et al. [5] suggested that the anti-MICA antibodies were formed in patients with no transfusion history in majority of their cases. They suggested the formation of these antibodies to the cross-reactivity with the substances from the environment, thereby priming the immune system and facilitating the anti-MICA antibody formation.

Our study had certain limitations. Only undiluted sera were used to perform the antibody assays; therefore, prozone phenomenon, if any, could not be ascertained. Typing of the donors for MICA antigens could not be performed due to limited resources; thus, formal proof of donor specificity could not be obtained. We attempted to investigate the role of transfusion as one of the sources of immunization. However, it would be interesting to examine pregnancy or a sensitizing event like previous transplants as sources of immunization.

The current consensus does not recommend routine testing for non-HLA antibodies prior to the transplant [30]. To conclude, we favor the hypothesis that anti-MICA antibodies are causally involved in allograft rejection. Preformed anti-MICA antibodies independently increase the risk of kidney rejection, and therefore we recommend that guidelines should be formed for mandatory testing of these antibodies prior to transplant as well as strategies to reduce or eliminate the effect of anti-MICA antibodies on kidney graft outcome.

## Figures and Tables

**Table 1:** Characteristics of the cohort A (*n* = 646) population.

Characteristic	MICA-negative group (*N* = 552)	MICA-positive group (*N* = 94)	*P* value
Geographic origin—number (%)			
Asia	389 (86.6)	69 (13.4)	0.20
Africa	163 (82.7)	34 (17.3)	
Recipient sex—number (%)			
Male	399 (85.6)	67 (14.4)	0.84
Female	153 (85.0)	27 (15)	
Recipient race—number/total number (%)			
Indian	231 (87.2)	34 (12.8)	0.30
Non-Indian	321 (84.3)	60 (15.7)	

**Table 2:** Effect of pretransplantation transfusions on antibodies against HLA class I, HLA class II, or MICA.

Antibodies	Transfusion		*P* value
	Yes	No	
HLA class I—number (%)			0.01
Negative	355 (58.4)	253 (41.6)	
Positive	33 (86.8)	5 (13.2)	
HLA class II—number (%)			0.0001
Negative	353 (58.5)	250 (41.5)	
Positive	38 (88.4)	5 (11.6)	
MICA—number (%)			0.63
Negative	68 (12.3)	484 (87.7)	
Positive	9 (9.6)	85 (90.4)	

**Table 3:** Comparison of anti-HLA and anti-MICA antibodies in cohort B (*n* = 392).

Characteristic	MICA-negative group (*N* = 341)	MICA-positive group (*N* = 51)	*P* value
Latest panel-reactive HLA-antibody value—number (%) 0.58			
0%	297 (87.4)	43 (12.6)	
>0%	44 (84.6)	8 (15.4)	

**Table 4:** Effect of MICA antibodies on renal allograft.

Group	Number of patients	^∗^Hazard ratio (95% CI)	*P* value
All patients	646	3.0701 (1.7132–5.517)	0.0002
HLA− A+B+DR mismatches			
0-1	47	6.8333 (0.4895–95.3880)	0.1530
2–4	204	0.8912 (0.3611–2.1996)	0.8027
5-6	53	6.8571 (0.1475–318.6982)	0.3256
Anti-HLA antibody value			
0%	340	2.7628 (1.1333–6.7353)	0.0254
>0%	52	1.3750 (01756–10.7644)	0.7616

^∗^Hazard ratios are calculated for transplant rejection in patients who had antibodies against MICA as compared with patients who did not.

**Table 5:** Effect of HLA-ABDR mismatches on anti-MICA antibody formation.

Characteristic	MICA-negative group (*N* = 341)	MICA-positive group (*N* = 51)	*P* value
HLA-A+B+DR mismatches—number/total number (%) 304			
0-1	41 (87.2)	6 (12.8)	0.93
2–4	177 (86.8)	27 (13.2)	
5-6	47 (88.7)	6 (11.3)	

**Table 6:** Details of MICA antibodies detected.

Number of MICA-positive antigens	Number of patients (*n* = 94)
15	1
10	2
8	1
7	5
6	3
5	9
4	5
3	11
2	22
1	35
